# Perspectives of young people with neuromotor disabilities on shared digital portals in paediatric rehabilitation: A descriptive–interpretive qualitative study

**DOI:** 10.1111/dmcn.70203

**Published:** 2026-02-15

**Authors:** Marietta Kersalé, Diane Paquin, Sylvain Brochard, Christèle Kandalaft, Emeline Edern, Marie‐Pascale Pomey, Quan Nha Hong, Christelle Pons

**Affiliations:** ^1^ W.Inn, Center for Innovation CHU Brest Brest France; ^2^ Department of Physical Medicine and Rehabilitation CHU Brest Brest France; ^3^ Faculty of Medicine and Health Sciences University of Brest (UBO) Brest France; ^4^ Laboratory of Medical Information Processing (LaTIM) INSERM UMR 1101 Brest France; ^5^ Department of Paediatric Physical Medicine and Rehabilitation Fondation Ildys Brest France; ^6^ Parent Partner in Research Paris France; ^7^ Patient Partner in Research Brest France; ^8^ Faculty of Medicine University of Montréal (UdeM) Montréal Canada; ^9^ Research Center of the University of Montréal Hospital (CHUM) Montréal Canada; ^10^ Center of Excellence for Partnership with Patients and the Public (CEPPP) Montréal Canada; ^11^ Center for Interdisciplinary Research in Rehabilitation of Greater Montréal (CRIR) Montréal Canada

## Abstract

**Aim:**

To explore how adolescents and young adults with neuromotor disabilities perceive shared digital health portals as tools to support self‐management of their rehabilitation and integrated care.

**Method:**

We conducted a descriptive–interpretive qualitative study using semi‐structured interviews with 24 participants aged 11 to 23 years undergoing rehabilitation. Participants were recruited nationally through patient and family associations and rehabilitation networks, using purposive maximum variation sampling. An interdisciplinary team analysed the data using a hybrid inductive–deductive thematic approach grounded in self‐determination theory.

**Results:**

Participants viewed portals as enablers of integrated rehabilitation, aligning interventions across health care, home, and school according to individual needs. Features supporting competence, such as progress tracking and tailored resources, were valued for balancing rehabilitation with daily life. To foster autonomy, participants emphasized involvement in rehabilitation decisions, personalized feedback, control over information sharing, and inclusive features. Portals were also seen as a means to strengthen professionals' understanding and enhance connections with families and peers. Priorities included accessibility, adaptable designs, and customizable privacy settings. Concerns involved guilt‐inducing feedback, excessive monitoring, and reduced in‐person interactions.

**Interpretation:**

Young people with disabilities see shared portals as valuable for supporting self‐determination and integrated care. Co‐designing flexible, inclusive, and trustworthy tools with young people may strengthen coordinated, empowering rehabilitation experiences.

## Graphical abstract

Graphical abstract illustrating the four key themes identified in this study. From the perspective of young people with neuromotor disabilities, shared digital portals could improve interprofessional coordination and promote competence, autonomy, and relatedness in rehabilitation care.

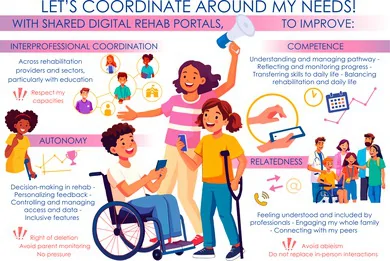

**This original article is commented by Nossell on pages** 1332 **of this issue.**

**What this paper adds**

- Portals could help coordinate rehabilitation and education, supporting continuity across settings in alignment with their needs.
- Participants expected features that support their ability to understand and manage their condition, reflect and monitor progress, and transfer skills to daily life.
- Young people valued autonomy through participatory goal setting, personalized and inclusive features, and control over access.
- Portals could support meaningful connections with professionals, family members, and peers.
- Young people cautioned against parental and professional monitoring, performance pressure, ableism, and reduced human connection.

Children and young people with childhood‐onset neuromotor disabilities often experience complex, long‐term impairments that require continuous, coordinated support across home, school, health care, and community settings.^1^, ^2^ Paediatric rehabilitation aims to optimize daily functioning, foster autonomy, and promote participation through multidisciplinary collaboration.^3^, ^4^ However, coordination across professionals, settings, and services remains challenging, particularly during key transitions, such as adolescence.^5^, ^6^, ^7^ Rehabilitation efforts frequently fall short of being truly centred on the needs and priorities of children and their families.^8^, ^9^ Fragmented rehabilitation systems place a significant burden on families,^10^ hinder effective communication among professionals,^5^, ^11^ and contribute to suboptimal health outcomes and reduced quality of life for children and young people with disabilities.^12^, ^13^

Therefore, improving communication about the functioning and participation of children and young people among all stakeholders, including caregivers and professionals in health care, education, and community settings, is critical for achieving better rehabilitation outcomes^4^, ^14^ and advancing integrated care.^15^, ^16^ However, to our knowledge, few solutions exist to address these gaps.

E‐health solutions, such as shared digital patient portals, have shown promise in enhancing communication, care coordination, self‐management, and patient engagement in the context of chronic conditions.^17^, ^18^, ^19^ These tools have the potential to address critical gaps in paediatric rehabilitation by facilitating continuous coordination across settings and services and empowering young people to take an active role in their rehabilitation journey. However, most existing digital portals are primarily focused on medical monitoring and treatment adherence,^20^, ^21^ and are often tailored to specific conditions,^22^ which limits their relevance for the broader needs of rehabilitation and cross‐sector collaboration.

Given the evolving nature of child and adolescent development, digital solutions should be flexible to respond to changing needs and promote meaningful engagement over time, through a life course approach. There is a critical need to develop user‐centred digital portals tailored to young people's rehabilitation experiences and responsive to their developmental trajectories.

To ensure these tools are effective and inclusive, it is essential to directly involve young people with disabilities in the design process.^23^ Without their engagement, digital innovations risk failing to meet real‐life needs, resulting in low uptake and exacerbating health inequalities.^24^

This work is part of a broader research project, Deneo Kid, conducted in France, which actively involves young people, families, and professionals^25^ to understand their needs and co‐design a shared digital health portal that supports integrated paediatric rehabilitation.

The guiding question of this study was: How could shared digital health portals improve integrated paediatric rehabilitation from the perspective of individuals with lived experience?

Accordingly, this study explored how adolescents and young adults with childhood‐onset neuromotor disabilities undergoing rehabilitation perceive the usefulness and functions of shared digital health portals as tools to support their self‐management of rehabilitation and integrated rehabilitation care. It specifically explored their needs, expectations, concerns, and desired features to inform the development of future digital tools.

## METHOD

### Study design

An interpretive–descriptive qualitative methodology was used to explore complex experiential questions and generate practical insights.^26^, ^27^ We adopted a constructivist epistemological approach, recognizing reality as multiple, contextual, and socially constructed, to generate meaning through a collaborative dialogue between researchers and participants.^28^ Our disciplinary orientation is grounded in rehabilitation sciences, with the overarching goal of improving rehabilitation services to support meaningful participation, which is consistent with the F‐words framework^8^ and patient and family partnership in care and research.^29^ The lead researcher's occupational science lens shaped the analysis, focusing on individuals' occupational balance and the transfer of rehabilitation activities into daily life environments. Complementary insights from disability sociology and lived‐experience partners were also integrated, enriching the analysis and ensuring that findings are both relevant to practice and informative for disciplinary knowledge. Semi‐structured individual interviews were chosen to gain an in‐depth understanding of each participant's rehabilitation journey^30^ and capture individualized perspectives on integrating digital tools into rehabilitation pathways.

### Participants and recruitment

Participants were eligible if they were aged 11 to 23 years, had a childhood‐onset disability,^1^, ^31^, ^32^ received paediatric rehabilitation, lived in France, and could engage verbally in an interview in French. They could be accompanied by a caregiver if needed. A purposive sampling strategy with maximum variation^33^ was used to capture diverse perspectives (Table 1). Key variation criteria were collected during the initial contact and reviewed by MK and CP to guide inclusion and ensure sample diversity. Recruitment took place from August 2024 to February 2025 through direct invitations from rehabilitation professionals within a paediatric rehabilitation network and flyers distributed on social media (LinkedIn, Facebook, Instagram). The sample size was determined pragmatically, in line with Thorne,^26^ with recruitment continuing until sufficient variation and depth of data had been obtained to allow meaningful and useful exploration of participants' experiences, while acknowledging that additional perspectives could provide further insight.

**TABLE 1:** Criteria used for the maximum variation sampling strategy.

Criterion	Details	Rationale
Age	11–23 years	To capture diverse perspectives across the transition from paediatric to adult rehabilitation
Childhood‐onset disability	Types of disabilities categorized using the International Classification of Diseases, 11th Revision,^34^ and the Diagnostic and Statistical Manual of Mental Disorders, Fifth Edition^35^	To represent a broad range of functional profiles and rehabilitation needs
Education and vocational status	Type of schooling, vocational training, or employment	To reflect diverse life trajectories and social disparities
Living situation	Independently or with other persons	To capture differences in autonomy and family involvement in daily life and care coordination
Rehabilitation setting	Participating in or having participated in paediatric rehabilitation in hospitals, centres, outpatient settings, or integrated health and social services	To reflect diverse care environments and service delivery models
Involvement in associations	Active or non‐active in disability‐related groups	To balance the views from individuals more or less engaged in advocacy and peer networks
Geographical region	Various regions across France	To account for regional disparities in service access

### Data collection

The interview guide was co‐designed with the research team and pre‐tested with a 19‐year‐old female with cerebral palsy (CP). Minor adjustments were made to the guide during the interview process. The final version included 10 open‐ended questions with prompts (Table 2). The explanation of shared portals and questions was adjusted in real time based on each participant's understanding. The interview invited participants to reflect broadly on the usefulness of shared digital portals and to freely imagine any features that could support their rehabilitation, allowing for open‐ended exploration of their needs, expectations, and ideas. At the end of the interview, to stimulate ideas about other potential features,^34^ participants were briefly introduced to Deneo via a live screen‐sharing demonstration.^35^ Deneo is a secure digital portal developed for adult sports rehabilitation, allowing both adult patients and professionals (mainly physicians, kinesiologists, and physiotherapists) to collaborate and monitor rehabilitation progress. A detailed description of the features is provided in Appendix S1.

**TABLE 2:** Interview guide.

Focus area	Sample interview questions
Ice breaker	What motivated you to participate in this interview?
Rehabilitation pathway	Can you tell me about your paediatric rehabilitation journey?^a^ *Prompts: Professionals, settings, transitions, collaboration with and between professionals, challenges, needs‐based care experience?*
Experience with digital tools	Have you ever used an app to manage your health or to communicate with professionals? Could you describe your experience?
Views on shared app for rehabilitation	What do you think of a shared app to manage your rehabilitation and communicate with professionals? *Prompt: How would it help you in your rehab?*
Access and information sharing	Who should have access to the app? *Prompts: Type of information to share or not share, your role, professionals' role, age to access, sharing with rehab/education/recreation professionals?*
Desired features	What features would you want included? Why?
Privacy and concerns	What concerns would you have about using such an app? *Prompts: Private information, safeguards, potential barriers?*
Feedback after the portal demonstration	What do you think of this app? *Prompts: Anything missing or that you would change? Useful features? Why?*
Use in daily life	How do you imagine using such an app in your daily routine? *Prompts: Which features would you use? In what situations?*
Future directions	Are there any additional features or recommendations you would suggest? *Prompt: Anything else you would like to add?*

^a^ Depending on their age and care trajectory, participants were prompted to discuss ongoing or past rehabilitation experiences, including intensive interventions or maintenance of abilities. Participants older than 20 years followed in adult services were invited to recall their paediatric rehabilitation journey.

Interviews were performed using Zoom Pro or in person at the most convenient place for participants. Two researchers conducted the interviews: MK, an occupational therapist and doctoral student in rehabilitation sciences experienced in qualitative research, and DP, a master's student in disability sociology. Neither had prior relationships with the participants. Interviews were audio‐recorded and were followed by a sociodemographic questionnaire (Table 3), which included a brief self‐rated digital literacy score on a 0 to 10 scale.

**TABLE 3:** Participant characteristics.

Characteristic	*n* (%)
Sex	
Male	13 (54)
Female	11 (46)
Age group, years	
11–14	6 (25)
15–18	5 (21)
19–23	13 (54)
Living situation	
With both parents	16 (67)
With one parent	2 (8)
Alone	6 (25)
Education/occupation	
Middle school (mainstream class)	3 (13)
Middle school (specialized class)	3 (13)
High school	4 (17)
Professional training	3 (13)
Undergraduate	7 (29)
Graduate	1 (4)
Seeking work	3 (13)
Involvement in disability‐related associations	
Yes	7 (29)
No	17 (71)
Health conditions	
Cerebral palsy	11 (46)
Attention‐deficit/hyperactivity disorder^a^	5 (21)
Specific learning disorder^a^	5 (21)
Developmental coordination disorder^a^	3 (13)
Autism^a^	2 (8)
Genetic anomaly	2 (8)
Neuromuscular disease	1 (4)
Rare congenital malformation	1 (4)
Amputation	1 (4)
Obesity^a^	1 (4)
Main or current rehabilitation setting	
Hospital setting	6 (25)
Integrated health and social care setting	6 (25)
Outpatient setting (including private practice)	12 (50)
Rehabilitation professionals involved^b^	
Occupational therapist	20 (83)
Physiotherapist	16 (67)
Psychologist (including neuropsychologist)	14 (58)
Psychomotor therapist	9 (38)
Kinesiologist	8 (33)
Speech and language therapist	7 (29)
Special needs educator	7 (29)
Orthoptist	2 (8)
Region of France	
Brittany	11 (46)
Occitanie	4 (17)
Nouvelle Aquitaine	2 (8)
Grand Est	1 (4)
Normandy	1 (4)
Bourgogne Franche‐Comté	1 (4)
Pays de la Loire	1 (4)
Centre‐Val de Loire	1 (4)
Paris region	1 (4)
Auvergne‐Rhône‐Alpes	1 (4)
Perceived digital literacy (0–10)^c^	Mean (SD) = 8.1 (1.5) Median (IQR) = 8.5 (1.3) Range = 4–10

Abbreviation: IQR, interquartile range.

^a^ Participants listed with these conditions presented at least two concurrent conditions.

^b^ All participants had from two to five rehabilitation professionals.

^c^ Digital literacy was self‐rated in the sociodemographic questionnaire in response to the question: ‘How would you describe your digital skills on a scale from 0 to 10?’ The scale ranged from 0 (‘no competence’) to 10 (‘full competence, such as downloading apps, sharing files, or using a new device independently’).

### Data analysis

Interviews were transcribed verbatim using artificial intelligence software^36^ and manually verified. Following Braun and Clarke's six‐step thematic analysis,^37^ MK and DP independently coded four interviews line by line and discussed codes to develop a codebook using NVivo14 (Lumivero, Denver, CO, USA). DP coded the remaining interviews, and regular meetings were held to discuss codes, construct and refine themes, and ensure sufficient variation and depth.

Preliminary themes were developed inductively. Because autonomy and motivation were identified as recurring patterns, the authors chose to use the basic psychological needs theory from self‐determination theory^38^ to organize and interpret the findings. Self‐determination theory highlights three core psychological needs: autonomy, competence, and relatedness. This framework provided a deeper understanding of how these needs can be addressed through digital tools in paediatric rehabilitation. Using this deductive analysis^39^ facilitated a nuanced analysis, connecting our findings to a well‐established motivational model, which has been used in previous studies exploring motivation in paediatric rehabilitation.^40^, ^41^

A constant comparative approach was used to examine patterns, properties, and dimensions within and across themes.^27^ This iterative process involved condensing excerpts from the transcribed interviews, comparing data within and between themes, and refining concepts to capture variations, relationships, and participants' meaning‐making.

To enhance credibility, participants were engaged strategically to refine the interpretations.^27^ Online group and individual sessions were conducted to review and discuss the preliminary themes and subthemes, illustrated with selected verbatim quotes, using open‐ended questions^42^ to assess accuracy, clarity, and completeness. Feedback from these sessions informed refinement of the final themes by all authors.

### Trustworthiness

For triangulation, an interdisciplinary team with expertise in rehabilitation, public health, sociology, and lived experience (a young adult with a childhood‐onset disability [EE], and a mother [CK]), contributed to interview guide development, recruitment, analysis, and manuscript revision.^43^ Field notes, reflexive journals, and regular team meetings supported data collection and analysis. Reflexive analysis was conducted individually and then collectively, guided by Patton's reflexive triangle,^44^ prompting researchers to examine how their culture, education, and values shaped their voice and interpretation of the results.^45^ Dependability was ensured through interviewer training, ongoing feedback, and reflexive self‐assessment. Reporting followed the Standards for Reporting Qualitative Research guidelines (Appendix S2).

### Ethical considerations

The study protocol was approved by the Territorial Ethics Reflection Committee of Brest University Hospital (ref. no. B2024CE.05). All participants received age‐appropriate information in easy‐to‐read formats. Informed consent was obtained orally from all participants, including for the use of their transcripts in publications and presentations. Parental consent was obtained orally or by e‐mail for minors. During manuscript preparation, participants were invited to choose their own pseudonyms; if they did not respond, no pseudonym was assigned. No financial compensation was offered. The procedures were registered at ClinicalTrials.gov under the identifier NCT06570148.

## RESULTS

Figure S1 presents participant enrolment. Between September 2024 and March 2025, 24 interviews were conducted (35–60 minutes; mean = 45), including six in‐person interviews at the hospital or rehabilitation centre, based on participant preference. Four participants chose to be accompanied (parent: *n* = 2; educator: *n* = 1; nurse: *n* = 1). After the first 15 interviews, recurring patterns and initial variations in experiences were identified. To explore additional perspectives and nuances in rehabilitation experiences and digital tool use, recruitment continued until 24 participants with varied profiles had been interviewed.

**FIGURE 1:**
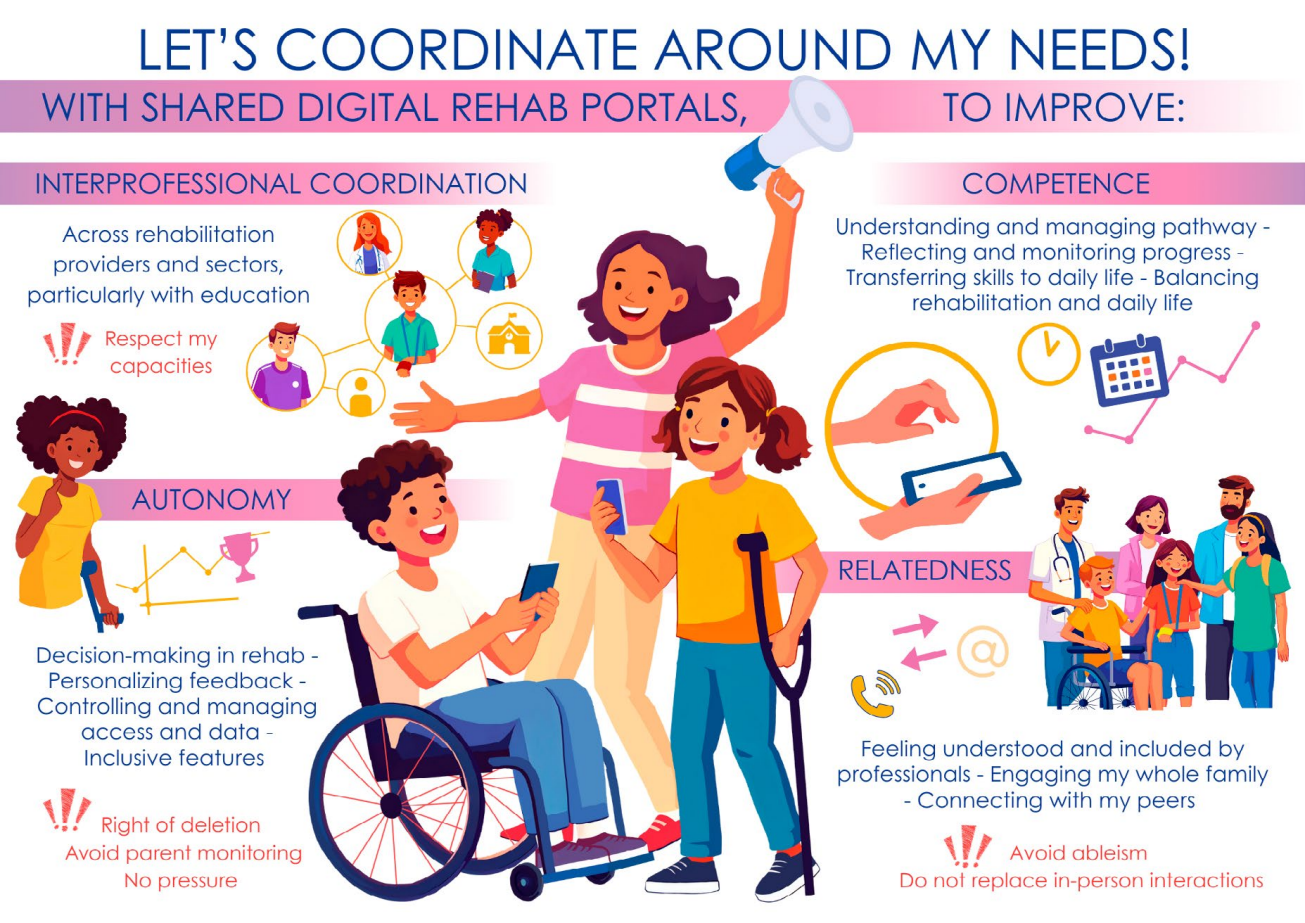
Mapping of key themes and subthemes.

Ten participants took part in two online groups and two individual sessions to review and discuss preliminary themes. All participants received the visual summary of the final results (Figure 1) by e‐mail, but none provided additional feedback.

Participants included 13 males and 11 females, with a median age of 19 years (range = 11–23 years), from 10 of the 13 regions of France. Most participants (*n* = 18) lived with one or two parents. Eighteen participants were students. Seven participants were actively engaged in disability‐related associations. The most common primary diagnoses were nervous system diseases (*n* = 12), followed by mental and neurodevelopmental disorders (*n =* 8), musculoskeletal diseases (*n =* 2), and developmental anomalies (*n* = 2). Most participants participated in rehabilitation in outpatient settings (*n* = 12), followed by hospital‐based services (*n =* 7) and integrated health and social services (*n* = 5). All participants reported daily smartphone use, with a median perceived digital literacy score of 8.5 out of 10 (Table 3).

Four themes were constructed and all framed to complete the sentence ‘I need a shared digital portal to …’: (1) improve professional coordination; (2) enhance my competence; (3) foster my autonomy; and (4) strengthen my relatedness. The first theme was developed through inductive analysis and the others were structured through deductive analysis with self‐determination theory. For each theme, participants discussed the portals' perceived usefulness, key features to support their needs, and critical safeguards. Figure 1 presents the themes and subthemes.

### Improving interprofessional coordination across settings and sectors (theme 1)

Interprofessional coordination refers to how professionals organize, communicate, and align their actions across settings to provide coherent and continuous support tailored to the child's needs.^14^ Two subthemes were identified: (1) coordinating rehabilitation across providers and (2) integrating rehabilitation and education into a unified life plan.

#### Coordinating rehabilitation across providers

Participants described specific components they considered critical for achieving coordination, particularly clear communication between providers and continuity of interventions across services and sectors. When these components were not present, care was experienced as fragmented, exhausting, and sometimes contradictory, highlighting how gaps in coordination can disrupt the coherence and effectiveness of rehabilitation.

Many participants emphasized feeling overwhelmed by the overall volume of rehabilitation. Poor communication between providers amplified this burden by creating unnecessary repetition, as illustrated by a 13‐year‐old female with CP, and by four different professionals:

Sometimes professionals do almost the same thing. My OT and my SLT both work on logical reasoning.

This overlap was interpreted as a lack of communication and shared understanding of goals, rather than reinforcement.

Shared digital portals were perceived as potential solutions to address these coordination gaps. Shared workspaces were seen as a way to clarify ‘what has already been worked on, so they don't redo the same thing’, as explained by an 11‐year‐old female with attention‐deficit/hyperactivity disorder (ADHD) and specific learning disorder. Features such as a ‘messaging system linked to each appointment’ were considered potentially helpful for improving communication about rehabilitation goals, particularly in outpatient settings, where coordination was perceived as weakest. However, some young people also noted that professionals have limited time for coordinating care.

Finally, participants associated better coordination through digital tools with improved quality of life. Reducing the need to repeatedly explain their situation and ensuring that professionals had access to the same information were seen as meaningful improvements that could save time and make the care pathway much simpler.

#### Integrating rehabilitation and education into a unified life plan

Participants also emphasized the need for better coordination across sectors, and particularly between rehabilitation and education. Communication gaps often led to inconsistent support for young people, particularly in outpatient settings. Vic, a 23‐year‐old with CP and mainly followed in outpatient settings, explained:

Few therapists attend school meetings, limiting opportunities to translate therapeutic recommendations into classroom adaptations, such as ‘workstation modifications’.

Digital portals were envisioned as tools to bridge these parallel systems, ensuring that all professionals remain informed and can provide personalized, responsive support. An 11‐year‐old female with ADHD illustrated this potential:

When I struggle with concentration, my teacher could inform my psychomotor therapist.

Participants also noted potential efficiency gains, particularly for multidisciplinary meetings, highlighting the time that could be saved if information was consolidated digitally.

However, some participants stressed the importance of contextualized interpretation. For example, a 13‐year‐old with CP in a mainstream class expressed concern that increased access to information might lead school staff to rigidly apply therapeutic expectations without considering fatigue or day‐to‐day variability:

If she says ‘you're capable of doing this with the OT’, but I'm tired at that moment … then I get blamed …

Overall, participants viewed portals as enablers of a more coordinated life plan and a more inclusive pathway, facilitating communication between rehabilitation professionals and with schools. At the same time, they emphasized the need to respect individual variability and to ensure that professional collaboration genuinely translates into meaningful, personalized support for young people.

### Enhancing my competence (theme 2)

Competence refers to an individual's belief in their ability to manage important tasks effectively.^40^ Participants described ways in which digital portals could support their competence, which were structured in four interrelated subthemes: (1) understanding and managing their condition and care pathway; (2) reflecting and monitoring progress; (3) transferring skills to daily life; and (4) balancing rehabilitation and daily life.

#### Understanding and managing their condition and care pathway

A key dimension of competence for young people with disabilities involves understanding their diagnoses, treatment options, available services, and rights, as well as the ability to interpret complex health information. When well developed, this competence allows young people to take responsibility for their health and make informed decisions. When limited, it can generate confusion, stress, and uncertainty about navigating the care system, as a 20‐year‐old male with developmental coordination disorder and with 15 years of rehabilitation experience reflected:

Sometimes we don't know where to go. It's really stressful.

Participants imagined that digital portals could support this competence by offering a centralized, accessible source of information about conditions and treatments, including artificial intelligence‐assisted simplification of clinical documents. Vic, a 23‐year‐old with CP, projected such a feature:

For my SLT assessment, using AI to highlight key points instead of reading five pages.

A growing understanding of their condition through digital tools was also envisioned as reassuring, providing a foundation for gradually assuming adult responsibilities. Arthur, a 17‐year‐old in his last year of high school, explained:

This app would help us move beyond adolescence … gradually realizing we'll have adult responsibilities. If we start taking them on now, things will go well.

#### Reflecting and monitoring progress

Well‐developed monitoring allows young people to recognize their progress, build confidence in their abilities, and maintain motivation, whereas its absence leads to uncertainty and dependence on professionals. Participants highlighted the importance of tracking, evaluating, and reflecting on their progress over time as key elements for developing competence and self‐management skills.

A 17‐year‐old male with CP, who had been in multidisciplinary rehabilitation since birth, illustrated this gap:

My PT saw my progress. It took me 7 years to notice mine.

Digital portals were perceived as practical tools to support this process by providing progress visualization, access to clinical reports, and milestone notifications. These features were described as fostering self‐reflection and strengthening awareness of achievements.

#### Transferring skills to daily life

Building on their developing understanding of their condition and progress, participants emphasized the importance of receiving clearer, more actionable guidance to transfer rehabilitation goals into everyday life, either independently or with family support. When this transfer is successful, young people can maintain or improve participation; when it is not, there is a risk of stagnation, regression, and frustration.

Jade, an 18‐year‐old with CP who had completed several intensive motor rehabilitation programmes, highlighted the importance of home support and continuity:

Sometimes we don't apply goals at home because it's too complicated and there's no real follow‐up.

Participants envisioned digital portals as tools to facilitate this transfer by providing practical resources, such as instructional videos, illustrations, or ways to contact professionals. Wyno, a 23‐year‐old with CP and expressing frustration with her rehabilitation, described the potential of this tool:

If I don't have PT, I'd know which exercises to do on my own or with my family's help. With videos or drawings.

#### Balancing rehabilitation and daily life

Participants, particularly when reflecting on their childhood, described demanding rehabilitation schedules that often required them to miss classes or sacrifice weekends, as a 20‐year‐old male with developmental coordination disorder and specific learning disorder recalled. These accounts highlighted that rehabilitation schedules were not always adapted to individual personal lives, making it difficult to balance therapy with school, leisure, and other daily activities. Gaëtan, a 22‐year‐old with specific learning disorder, reflected on the cumulative overload when recalling earlier periods of intensive therapy:

There's no point in making kids see five professionals weekly. They need a childhood.

Competence in this domain involves anticipating energy demands and managing time to maintain a functional balance across multiple life domains.

Digital portals, through interoperable calendars and reminders, were envisioned as practical tools to support this balance, helping children and young people prepare for sessions both physically and mentally, and organize their routines accordingly. A 13‐year‐old with ADHD followed by three different therapists explained:

When I have multiple appointments, I get lost. Having reminders would help me anticipate better.

### Fostering my autonomy (theme 3)

Autonomy refers to a person's ability to make choices, exercise control over their life, and act according to their values and preferences.^40^ Participants described ways in which digital portals could foster autonomy, structured around four interrelated subthemes: (1) decision‐making in rehabilitation; (2) personalizing goal‐based feedback; (3) controlling access and managing data; and (4) accessible and inclusive features.

#### Decision‐making in rehabilitation

Autonomy in rehabilitation involves having meaningful decision‐making power as active partners, rather than passively following professional instructions.

For participants, this often meant being involved in selecting their rehabilitation goals. Not having this control could lead to frustration and disengagement. Wyno, a 23‐year‐old who had spent several years in a rehabilitation centre, explained how using a portal to set her goals could help:

To put down my goals and really focus on them, I don't want to be all over the place. Once, my PT didn't work on any of them. It was frustrating.

Participants also stressed the importance of being able to decide whether to continue or stop rehabilitation, reinforcing the link between autonomy and their sense of competence. As Vic, a 23‐year‐old with CP and pursuing a master's degree, noted:

It would have helped when I decided to stop rehabilitation at 17. To know whether it was really the right choice… [laugh].

These reflections suggest a potential focus for digital portals, that is, supporting adolescents during the transition to adult services, a period when many may discontinue rehabilitation. Portals could provide a guide to facilitate shared decision‐making, helping young people navigate these critical choices while maintaining control over their rehabilitation.

#### Personalizing goal‐based feedback

Building on their competence to monitor progress, several young people described the ability to personalize rewards as a way to foster autonomy and engagement with rehabilitation goals. Digital portals were seen as tools to support this process through visual progress tracking and motivating feedback, such as colour‐coded circles or stars. Sylvain, a 20‐year‐old actively seeking ways to stay motivated in his rehabilitation, illustrated this feature:

Having a list of goals that professionals regularly update, with colour‐coded circles, green, orange …, and rewards. Like, ‘now, he showers on his own’.

Importantly, although personalized rewards were generally valued, participants emphasized the importance of avoiding pressure, frustration, or overly directive guidance. A 19‐year‐old male with CP, involved in disability‐related associations, cautioned:

The app shouldn't keep spamming reminders if someone can't reach a goal because of setbacks.

Similarly, Gabbro, a 22‐year‐old autistic male, stressed that excessive guidance could be counterproductive:

I wouldn't like a supervisor on my phone telling me what to do, forcing me or giving me rules … it could be more of a handicap than anything else.

Together, these perspectives highlight that digital portals should guide and motivate, while ultimately allowing young people to control how they use them and make their own choices.

#### Controlling access and managing data

A key dimension of autonomy for participants was the ability to choose who can access their information on portals. When well developed, this sense of control allows young people to feel secure, respected, and empowered; when limited, it can lead to discomfort or a feeling of being over‐monitored.

Participants emphasized the importance of customizable, selective access based on the type of information and the type of provider, particularly for school or recreation staff, with divergent views on who should have access. For example, Sylvain, a 20‐year‐old involved in a sports association, explained:

I know I'll get on well with my adapted sports coach. But I wouldn't want my school to know how my morale is.

Long‐term control, such as the ability to delete outdated content, was another core property. Leïla, an 18‐year‐old describing herself as very active on social media, reflected:

I'm afraid I'll make a mistake, like posting a video that I find good, but when I'm 25, I may want to remove it.

Parental oversight was also critical. Participants valued graduated control, allowing them to progressively manage the information shared during the transition to adulthood. Arthur, a 17‐year‐old adolescent who valued autonomy, stated:

From 16, we should have the freedom to decide what to share with health care professionals.

Wyno added:

Parents would want to check in all the time … I wouldn't want them to have access to everything. We should decide what they can see.

Participants suggested that digital portals could support autonomy by enabling tiered access, time‐limited sharing, deletion of outdated content, and transparent consent management, helping young people exercise control over their data while gradually assuming responsibility.

#### Accessible and inclusive features

A key aspect of autonomy is having digital portals accessible to all types of disabilities, enabling participants to engage independently. Participants highlighted the importance of universal design features, such as eye tracking, voice transcription, and text to speech for visually impaired users, as Leïla, an 18‐year‐old with CP involved in disability‐related associations, noted.

Gaëtan, a 22‐year‐old with specific learning disorder and ADHD, also involved in disability‐related associations, stressed the importance of alternative tools for those struggling to express themselves:

A small sun icon could indicate well‐being and fatigue levels.

Thus, digital portals were seen as tools that support autonomous expression, allowing young people to communicate their needs safely, adaptively, and inclusively, ultimately enhancing engagement and self‐determination.

### Strengthening my relatedness (theme 4)

Relatedness refers to feeling connected to others, cared for, and experiencing meaningful relationships.^40^ Participants described ways in which digital portals could reinforce connections with (1) professionals, (2) families, and (3) peers.

#### Feeling understood and included by professionals

Building on the idea of providing autonomy through inclusive features, participants described how expressing themselves via digital tools could help communicate fatigue, pain, or other constraints on their own terms. This self‐expression was considered important for helping professionals to understand their daily challenges and provide support tailored to their needs. Arthur, a 17‐year‐old with a rare congenital malformation, explained:

Giving young people a space to express themselves … If we can share how we feel, physically or mentally, they would understand … It would help them adapt.

Arthur also emphasized the importance of inclusion in educational settings, ensuring respectful, stigma‐free awareness of young people's challenges. Feeling recognized and included by professionals was central for him, with a caution against ableist attitudes:

We're caught in a cycle: rehab, home, school. They must understand it's not easy. Not victimization. We're like everyone else, but with an extra burden that should be considered. […] To truly include the pupil, isn't saying ‘respect your little disabled friend and help him’.

Participants suggested simple, personalized tools such as gauges, emoticons, or short comments to indicate well‐being or fatigue and selectively communicate this with professionals, as Eva, a 20‐year‐old with CP, illustrated:

A simple scale, like ‘I feel great today’ or ‘I feel exhausted’, with the option to add a comment and decide whether to share it.

Additionally, a 17‐year‐old describing himself as not very comfortable with digital formats, cautioned against relying solely on digital tools for communication with professionals:

To communicate with someone, I'd rather not depend entirely on the app.

Overall, participants emphasized that digital portals could help young people feel understood and included by professionals, provided that ableist attitudes are not reinforced and real‐life interactions are not replaced.

#### Engaging my whole family to feel supported

A key aspect of relatedness for young people involves family understanding and involvement. When this support is present, they feel cared for and connected; when it is lacking, they may experience isolation or misunderstanding.

Leïla, an 18‐year‐old living part‐time in a health and social care facility, suggested a shared space for relatives to both follow progress and provide encouragement:

A family access feature, so my cousins could see my progress … with private groups for my cousins and parents.

Structured updates were also considered important to compensate for limited direct contact between parents and professionals. A 17‐year‐old male with CP, followed in outpatient settings, explained:

Parents and rehabilitation professionals only see each other for 5 minutes. A report for the parents would help them see progress.

Participants envisioned digital portals supporting family engagement through private shared spaces, progress reports, and notifications, fostering understanding and meaningful involvement, and ultimately helping to motivate the young person in their rehabilitation.

#### Connecting with my peers

Finally, participants highlighted the importance of peer connections for social support, shared experiences, and reducing feelings of isolation. For young people with disabilities, well‐facilitated peer networks can foster a sense of belonging, whereas a lack of connection can reinforce isolation.

A 17‐year‐old female with a rare neuromuscular disease proposed:

A chat to share our lives with people our age … like a social network, but for young people with disabilities.

Participants envisioned digital portals that support peer relatedness through opt‐in social features, moderated groups, and progress‐sharing functions, enabling meaningful and motivating interactions that could both strengthen existing peer relationships and foster new connections by sharing achievements, progress, and sources of motivation.

## DISCUSSION

To our knowledge, this is the first study to explore how young people with disabilities perceive shared rehabilitation portals from an integrated care perspective. Findings suggest that such tools may promote self‐determination by addressing fundamental psychological needs, such as competence, autonomy, and relatedness across rehabilitation, school, and home settings. Participants identified features such as shared calendars, goal setting, progress tracking, emotional expression tools, and peer connectivity as valuable supports for daily engagement in rehabilitation and life. These findings extend beyond the focus on motivation in previous self‐determination theory‐based rehabilitation studies,^40^, ^41^ highlighting how shared digital portals could support young people's abilities to make informed decisions about rehabilitation, including enabling self‐management strategies to better balance rehabilitation with daily life and deciding when to continue or pause therapy, while fostering meaningful relationships with professionals, family, and peers. However, some participants raised concerns about data management, potential pressure, the risk of ableism, and threats to autonomy, highlighting systemic challenges to be addressed to ensure that these tools truly enhance integrated care.

A key contribution of this study is the identification of portals as potential enablers of integrated rehabilitation, aligning interventions across settings and sectors according to young people's needs. This continuity across health care contexts, home, and school is essential for supporting participation and is a recognized goal of long‐term rehabilitation outcomes.^4^ At the same time, participants highlighted important implementation barriers. In particular, they reported that digital portals might increase professionals' workloads, unless accompanied by a genuine shift towards inclusive, holistic, and collaborative practices. Coordination gaps were especially noted in outpatient settings and interactions between rehabilitation and educational professionals, reflecting known issues in France.^46^ Addressing these challenges requires not only technical solutions but also a transformation of interprofessional collaboration, including rethinking power dynamics between professionals,^47^ and allocating dedicated, compensated time for coordination.

Contrary to much of the literature on patient health portals,^20^, ^48^, ^49^ participants' concerns regarding data security and confidentiality took a different form. Instead of general worries about breaches or unauthorized access by non‐health care professionals,^25^ they focused on controlling who could access their information and managing their past data, with trust and inclusivity in professional relationships shaping their willingness to share information. This suggests that young people with disabilities adopt a more relational view of privacy than is commonly assumed in patient portal literature. Nonetheless, some participants expressed worries that digital portals might serve as surveillance tools for professionals or parents, thus compromising the autonomy they are meant to promote. Many called for customizable access controls, including options to limit parental visibility. This desire conflicts with the French legal framework on health data, which grants parents access to their child's medical information until the age of 18 years.^50^ However, many of the features young people wish to use, such as calendars, goals, emotions, or photos, involve personal rather than strictly medical data and may not legally require parental access. At the same time, exercising digital autonomy requires cognitive maturity and cybersecurity awareness,^51^ raising important questions about when and how young people can meaningfully manage their data. These tensions highlight the need for a gradual, developmentally sensitive approach to digital consent that aligns legal and ethical considerations.^52^

Participants' interest in customizable features, such as participatory goal setting, emotional expression, and peer interaction, are dimensions often overlooked in gamification apps.^53^, ^54^ Furthermore, rehabilitation apps often focus on exercise and performance tracking,^55^ neglecting the emotional aspects that participants considered crucial for personalized support. Given young people's digital fluency, tools like emojis or chat‐based features may serve as meaningful bridges for communication with professionals. However, one participant cautioned against the risk of replacing in‐person contact, underscoring that such tools should complement, not substitute, direct interactions.^56^ Others expressed concerns that, despite the motivational potential of portals, reminders, or notifications about unmet goals could trigger feelings of pressure, guilt, or judgment, giving the tool a punitive rather than supportive role. This finding echoes previous work on the risks of digital surveillance^57^ and is particularly important for young people with disabilities, whose capacity and energy fluctuate with time, symptoms, and context. These insights highlight the need to co‐design feedback mechanisms that are flexible, contextualized, and personalized. This process must go beyond interface design to include a collaborative dialogue with users, ideally before using the portal, to define who interacts with whom, what is shared, when, and how, including choices around gamification elements such as awards and notifications, while taking individual variability and personal capacities into account.

This study has certain limitations. The sample, although diverse and appropriate in size for an exploratory design, overrepresents individuals with neurological conditions and underrepresents those with non‐communicative disabilities, a group frequently excluded from qualitative research.^58^ This is partly because rehabilitation services in France are predominantly focused on neuromotor impairments. Participants' rehabilitation experiences also varied widely, from ongoing intensive interventions to maintenance of abilities; for some, accounts included retrospective reflections on past rehabilitation, so some findings may be more applicable to certain trajectories than others. The voluntary nature and online participation may have introduced selection bias, favouring individuals with a positive disposition towards digital tools. The specificities of the French health care and education systems might limit the transferability of the findings.^59^ However, a key strength lies in the involvement of individuals with lived experience (EE, CK) in the analysis, who reviewed transcripts, challenged assumptions, and validated themes. Their input helped ground the findings in real‐life experiences and refine key concepts such as autonomy and user–professional balance. Future research should explore the perspectives of a larger and more diverse sample of children and adolescents with various types of disabilities, taking into account linguistic diversity and cultural differences.

### Conclusions

This study highlighted the potential of shared rehabilitation portals to enhance continuity and coordination across settings and sectors, grounded in the needs of competence, autonomy, and relatedness of children with disabilities. By providing structured tools for daily management, supporting decision‐making and shared plans through participatory goal setting and personalized features, and strengthening meaningful connections with professionals, families, and peers, digital portals may significantly improve the rehabilitation experience. Notably, promoting autonomy, that is, enabling young people to make informed choices and manage their rehabilitation care, was identified as a central benefit. Respecting young people's evolving capacities, rights, and needs for accessible design remains both essential and challenging for successful development. Personalization, gamification, and inclusivity are key to ensuring relevance and usability. Further research is needed to evaluate the effectiveness and implementation of shared portals in real‐world settings.

## Supporting information


**Figure S1:** Flow chart of participant enrolment.

**Appendix S1:** Overview of the Deneo adult portal.

**Appendix S2:** Standards for Reporting Qualitative Research (SRQR) guidelines.

## Data Availability

The data that support the findings of this study are available on request from the corresponding author. The data are not publicly available due to privacy or ethical restrictions.
