# Supplementary material for: Perspectives of young people with neuromotor disabilities on shared digital portals in paediatric rehabilitation: A descriptive–interpretive qualitative study
Source: Dev Med Child Neurol. 2026 Feb 15;68(10):1398–411. doi: 10.1111/dmcn.70203 (PMC13545474; doi:10.1111/dmcn.70203)
Supplement: Supplementary file 1 [file DMCN-68-1398-s001.docx]

**Appendix 1. Overview of the Deneo Adult Portal**

Deneo (<https://deneo.fr/>) is a secure, multi-function shared digital portal designed for health management, initially developed in 2020 for adult sports rehabilitation and currently primarily deployed in French Sport and Health Centers (“Maisons Sport Santé”). Accessible via web and mobile app, the portal was developed using an agile methodology with iterative improvements based on user feedback. It allows both adults and healthcare professionals (mainly physicians, kinesiologists, and physiotherapists) to collaborate and monitor rehabilitation progress while complying with GDPR and storing data on certified French HDS servers. The adult portal is not linked to electronic health/medical records (EHR/EMR). However, future paediatric versions may include integration with records used across healthcare, integrated health and social care, and outpatient settings in France that provide rehabilitation care for children with neuromotor disabilities.

To our knowledge, no other similar shared portals exist in France for rehabilitation, which is why this adult portal served as a conceptual starting point for developing a shared paediatric portal for the Deneo Kid project. During interviews, researchers briefly presented the adult portal to stimulate participants’ ideas about potential features. Nevertheless, the paediatric portal will be fundamentally shaped by adolescents’ and young adults’ needs and adapted in future versions.

**Table A1. Key features and functions of the Deneo adult portal.**

| Feature | Key Characteristics / Functions | Interaction / Engagement |
| --- | --- | --- |
| Access / Permissions | Fine-grained management of access rights for patients and professionals. Ability to customize visibility of documents, programs, and feedback. A caregiver space is under development for the adult version. Secure login required. | Patients and professionals actively define who can see each item. Users manage their own sharing settings. |
| Dashboard / Shared calendar | Displays recently generated documents, at-home exercise programs, and a shared calendar. Notifications can alert users to new programs or documents (via email/SMS). | Patients view tasks, mark exercises as completed, check upcoming activities in the shared calendar, and respond to alerts. Professionals supervise, adjust programs, and send updates. |
| History | Stores all internal documents (assessments, tests, evaluations) and external files (PDFs, videos, multimedia). Users can view, download, comment, and annotate. | Patients can view and complete documents, add comments. Professionals generate, validate, and annotate documents. |
| Progress / Evolution | Visualizes progress through graphs based on assessments and tests, allowing longitudinal tracking. | Patients view personal progress graphs. Professionals analyze data to adjust follow-up plans. |
| Follow-up / Orientation | Supports referral to partner facilities with an interactive map of Sport Santé facilities. | Patients explore options and recommendations. Professionals guide patients and share information with partner centers. |
| Programming / Exercise Plans | Create and monitor personalized exercise programs, including multimedia (images, videos, YouTube links), templates, and feedback (pain scales, Borg…). Programs are linked to the shared calendar. | Patients complete exercises and provide feedback. Professionals create, personalize, and adjust programs in real time. |
| Document Generation | Generates internal documents (validated assessments or custom reports) and external files (e.g., medical PDFs). Visualizes progress via charts. | Patients generate personal documents where applicable. Professionals create assessments, send documents, and analyze patient responses. |
| Notifications / Alerts | Custom alerts for new exercises, programs, or documents. Notifications via email or SMS. | Patients receive alerts and act (view, complete exercises, provide feedback). Professionals configure alerts and monitor engagement. |

The corresponding screenshots (A1–A6) illustrate the key features and functions summarized in Table A1, with each screenshot corresponding to a specific feature listed in the table.

**Screenshots A1–A6. Screenshots of the Deneo adult portal shown to participants during interviews.**

*For demonstration purposes, a fictitious child profile was created, but the screenshots reflect the actual functionalities of the adult version of the portal.*

Screenshot A1. Navigation menu tabs accessible to patients and professionals (Dashboard, History, Evolution, Orientation, and Programming).

Screenshot A2. Dashboard tab – shared calendar view for patients and professionals

Screenshot A3. History tab – list of documents generated throughout the patient's care pathway, organized by month and year (e.g., assessments created within the platform and external medical reports).

Screenshot A4. Progress tab – graphical visualization of assessment results (Example: measurement of motor function)

Screenshot A5. Orientation tab – interactive map of Sport Santé partner facilities in France.

Screenshot A6. Programming tab – personalized exercise plan with embedded YouTube video and difficulty monitoring (Example: tying shoes)
